# The Role of Rendering in the Competence Project in Measurement Science for Optical Reflection and Scattering

**DOI:** 10.6028/jres.107.020

**Published:** 2002-06-01

**Authors:** Harold B. Westlund, Gary W. Meyer, Fern Y. Hunt

**Affiliations:** Department of Computer and Information Science, University of Oregon, Eugene, Oregon 97403; National Institute of Standards and Technology, Gaithersburg, MD 20899-8910

**Keywords:** BRDF, computer rendering, gloss, reflectance

## Abstract

Computer rendering is used to simulate the appearance of lighted objects for applications in architectural design, for animation and simulation in the entertainment industry, and for display and design in the automobile industry. Rapid advances in computer graphics technology suggest that in the near future it will be possible to produce photorealistic images of coated surfaces from scattering data. This could enable the identification of important parameters in the coatings manufacturing process that lead to desirable appearance, and to the design of virtual surfaces by visualizing prospective coating formulations once their optical properties are known. Here we report the results of our work to produce visually and radiometrically accurate renderings of selected appearance attributes of sample coated surfaces. It required changes in the rendering programs, which in general are not designed to accept high quality optical and material measurements, and changes in the optical measurement protocols. An outcome of this research is that some current ASTM standards can be replaced or enhanced by computer based standards of appearance.

## 1. Introduction and Background

The durability and attractiveness of paint and related polymer coatings are crucial to the marketability and performance of manufactured products from virtually every industrial sector: automobiles, computer screens, buildings and home appliances to name a few. As advances in material science and technology enhance our ability to manufacture coatings with novel and attractive visual effects, customer expectations for these products have increased. Thus maintaining the consistency and predictability of surface coating performance in this environment will require that companies have the ability to predict appearance at the coatings formulation level—the level most under the manufacturer’s control. Progress in material characterization and optical metrology are the foundation for achieving this capability, but rapid advances in information technology suggest that it will be possible in the near future to use the results of optical and material measurements to render the appearance of painted surfaces from which the measurements were taken. Computer graphic rendering is used to simulate the appearance of lighted objects for design applications in architecture, for animation and simulation in the entertainment industry, and for display and design in the automobile industry.

In the future, rendering could be used to predict the appearance of a surface from the optical properties of the coating, permitting the development of tools that can be used firstly to identify important parameters in the formulation process and secondly to allow designers to visualize the appearance of a proposed coating. The ability to view a virtual end stage product will eventually lead to a computer graphics based standard for appearance. In this report we will describe some first steps toward this goal. The work reported here was part of an effort arising from a competency project entitled “Measurement Science for Optical Reflectance and Scattering” involving four laboratories at NIST: the Building and Fire Research Laboratory, the Information Technology Laboratory, the Manufacturing Engineering Laboratory, and the Physics Laboratory. The project was initiated in response to recommendations by industry [1] and the Council of Optical Radiation Measurements [2]. Its purpose was to apply the technical advances in material science, optical metrology, light scattering modeling and computer graphic rendering to develop more accurate methods of modeling and predicting the appearance of coatings and coated objects.

### 1.1 The Physical and Quantitative Characterization of Appearance

When we use the term “appearance” we are referring to a complex of attributes determined by the interactions of light with a surface. These include but are not limited to gloss, glitter, color and fluorescence. We will not discuss the human psychophysics involved in the perception of these characteristics, although rendering programs do take these considerations into account and investigation of their effects is an active area of research [3]. When you look at an object, say a tabletop in a lighted room lit by a single lightsource which for simplicity we assume has a single wavelength, the amount of light that reaches your eye from the table can be derived from a function whose values are the fraction of light incident on the surface that is scattered in the direction of your line of sight. This function is known as the bi-directional reflectance distribution function (BRDF). Each surface has a BRDF that quantitatively summarizes its light scattering characteristics. It is an assumption in our research that knowledge of the BRDF is essential to successful representation of surface appearance. This assumption was strongly supported in a number of meetings between NIST members of the competency project and researchers from industry and academia concerned with appearance including NIST Workshops held in 1996 and 2000 and at SIGGRAPH meetings in 1997 and 1999.

### 1.2 Rendering

In order to realize the overall purposes of the project that we mentioned at the beginning, we set the goal of producing visually and radiometrically accurate renderings of selected aspects of the appearance of complex surfaces; seeking to provide a proven path from material measurement and characterization to object rendering. At this point, it would be helpful to answer the question, just what is rendering? It is the process of producing a synthetic image using a computer. To do this, certain input parameters are required. If the scene is a conference room for example, a geometric description of the objects in the room (furniture, light sources, carpeting, windows, etc.) must be provided. Second, there needs to be a description of the light sources, their location and radiometric properties. Third, the light scattering properties of an object in the room must be described. Thus we must have the BRDF of the object or BRTDF if light is also transmitted. Finally an observation viewpoint must be specified that defines the plane from which the image will be viewed. These parameters are used in an integral equation which describes the relationship between the amount of light incident to a surface in every direction and at a given point, and the amount of light reflected or emitted from the surface in a given direction at that point.

$$\begin{array}{c} L_{r}\left(\theta_{r},\phi_{r};\lambda ;x,y,z\right)=\int_{\omega_{i}}\rho L_{i}\left(\theta_{i},\phi_{i};\lambda ;x,y,z\right)\\ \cos\left(\theta_{i}\right)d\omega_{i}+\text{emittance} \end{array}$$ (1)

where *ρ* = *ρ*(*θ*_i_, *ϕ*_i_; *θ*_r_, *ϕ*_r_; *λ*) is the BRDF of a surface at the incident direction *θ*_i_, *ϕ*_i_ and reflected direction *θ*_r_, *ϕ*_r_, and *λ* is the wavelength of the incident light which we assume remains the same when reflected. *L*_i_ (*θ*_i_, *ϕ*_i_; *λ*; *x, y, z*) is the radiance of incoming light at (*x,y,z*) travelling in the direction given by *θ*_i_, *ϕ*_i_. *L*_r_(*θ*_r_, *ϕ*_r_; *λ*; *x, y, z*) is similarly defined for light in an outgoing direction from (*x, y, z*) [4]. The units of radiance are W/m^2^sr where sr (steradians) are solid angle units [5]. The radiance of light incident at a point (*x, y, z*) can be related to the outgoing radiance at the same point so that the integral equation can be written as an equation for a single unknown radiance function *L* [4]. A large part of the work done by the rendering program is the computation of a numerical solution of this equation, providing the radiance for each point in the scene that is visible to the observer or detector. A visual representation of the solution in a geometric description of the scene is the basis for the synthetic image. Global illumination methods [4] compute the contribution to the radiance at a point coming from direct illumination (i.e., incident light coming directly from light sources) and the contribution from indirect illumination, that is light that has been scattered and reflected off other surfaces in the scene before reaching the point. Physics based methods attempt to accurately describe the propagation of light and its interaction with materials in the scene, often using results from the fields of radiative heat transfer and illumination engineering. Diffraction, polarization and other effects that depend on the wave-like nature of light are taken into account to some extent, but computations are based primarily on geometric optics. Approximations to the BRDF used in these rendering programs have been developed largely in isolation from the BRDF measurement and modeling community. Because the major rendering applications have come from the entertainment industry, speed has been more important than physical accuracy. Our first task, therefore, was to build an interface from the high precision BRDF measurements and models to forms suitable for input to a rendering program. As a starting point we used the program Radiance discussed in the next section. The result was an enhanced rendering program called iBRDF (see Sec. 4)—a significant step towards achieving project goals.

### 1.3 Radiance and NIST Measurements

Radiance is a publicly available and widely used rendering program which is physically accurate and which uses global illumination methods [6]. We worked with its author, Gregory Ward, to determine requirements for the interface and with Michael Metzler, who wrote the modeling and data fitting code for the NEFDS [7] database that we will discuss later. We sought to answer the question, what kind of BRDF data is needed for rendering? Like many rendering programs, Radiance uses Monte Carlo methods to evaluate the integral on the right hand side of the radiance equation. In order to assess the contribution of light from points in the scene to light reaching the observer, many values of the BRDF of the surface containing the contributing point must be used, particularly when light coming from diffuse interreflections is computed. To meet this need, one might consider exhaustive measurements. However for diffuse materials these are unnecessary and specular materials would require extensive sampling that would be too time consuming and difficult to be feasible. Our approach—a mixture of modelling and measurements was tailored to the appearance attributes and materials under study with the aim of balancing physical accuracy and computational efficiency. Two materials are discussed here. Black glass samples with a clear epoxy coating and metal panels painted with metallic paint. Gloss was studied in the black glass samples and gloss, haze, and color were investigated in the metallic painted panels. In the case of black glass, a reflection model based on the geometric ray approximation of light propagation and on surface topographical measurements produced a BRDF of adequate accuracy. Large numbers of BRDF values could be generated easily and were used as input for iBRDF. A modified Beard-Maxwell model [8] was used for the painted panels. Parameters for the model were determined from reflectance measurements using a protocol like that employed in the NEFDS database.

### 1.4 Evaluation and Comparison

Rendering simulates a human observer looking at a real scene by converting reflectance and spectral characteristics into perceptual characteristics. What metrics can be used to evaluate how well a conversion method works especially in comparison with other methods? Since visual inspection is an irreducible part of any comparison, a rigorous quantitative metric must include an adequate model of human perception and psychophysics. Since the considerations leading to such a model were beyond our scope we were forced to rely on visual comparison of an object and its rendered image combined with a comparison of radiance measurements of the object and image. This was done for objects in a light box illuminated by light sources of known radiometric characteristics. The results of this study can be found in [9].

In the next section we describe the leading BRDF models used in rendering and a tool specially developed for visualizing them—the Oregon BRDF Library. A description of iBRDF is at the beginning of Sec. 4 and is followed by a description of its use in rendering the appearance of the black glass samples and painted panels. ASTM standards can be tied to the parameters of BRDF rendering models. This is discussed in Sec. 4.2. A discussion of the implications of these results for computer based appearance standards can be found in the concluding section.

## 2. Oregon BRDF Library

The appearance of an object is determined by the interaction of light with the object surface; the wide variation in the appearance of objects that we see can be linked to the variation in the light-surface interaction. This light-surface interaction is generalized by the bidirectional reflectance distribution function, BRDF, and has been studied extensively in many fields of research. The Oregon BRDF Library, part of masters thesis work reported in [10] (OBL) is a compilation of a wide range of BRDF functions constructed over the previous 30 years in the fields of physics and computer graphics. OBL offers a uniform, object oriented interface in C++ to this collection of BRDF functions, and is flexible enough to incorporate future BRDF models.

**Fig. 1:**
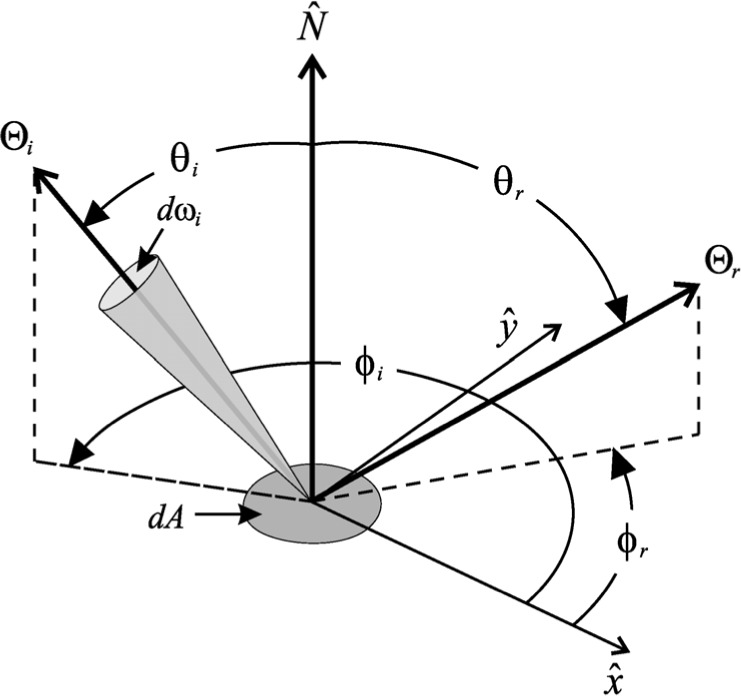
Light reflection geometry.

Each BRDF included in OBL is based on the following definition. The BRDF of a surface, *ρ*, is the ratio of directionally exitant radiance to directionally incident irradiance at a particular wavelength:

$$\rho \left(\Theta_{i};\Theta_{r};\lambda \right)=\frac{dL_{r}\left(\Theta_{i};\Theta_{r};\lambda \right)}{dE_{i}\left(\Theta_{i};\lambda \right)}$$ (2)

where the subscripts i and r denote incident and reflected respectively, *Θ* = *θ*, *ϕ* is the direction of light propagation, *λ* is the wavelength of light, *L* is radiance, and *E* is irradiance [5]. The geometry used by the BRDF is shown in Fig. 1. OBL includes quite a diversity of BRDFs. They range from the simple to the complex and include both analytical and empirical models.

**Fig. 2:**
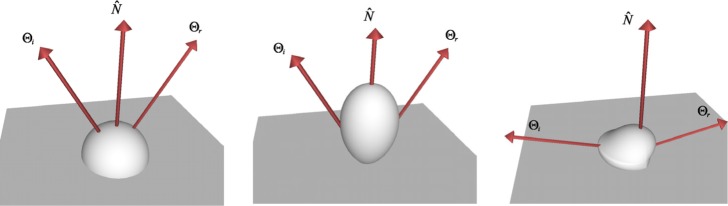
Diffuse BRDF models: Lambert, Minnaert and Oren-Nayar.

One of the most common and by far simplest BRDF provided by OBL is the one proposed by Lambert [11]. This is the BRDF of constant value (having constant radiance in all directions) which describes the reflection properties of a diffuse surface. Other more complex diffuse BRDF models are also included in OBL. Minnaert proposed a generalization of the Lambertian BRDF in order to describe the reflection properties of the moon [12]. Ideal diffuse reflector microfacets were used by Oren and Nayar to derive a physics based BRDF for diffuse surfaces [13]. These three diffuse OBL BRDF models (shown in Fig. 2) range widely in both their complexity and generality.

**Fig. 3:**
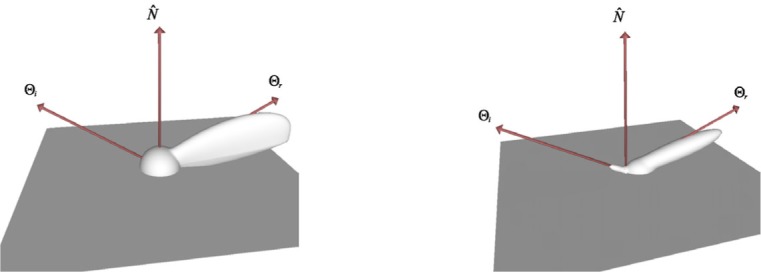
Spectrally variant BRDF models: Cook-Torrance and Lafortune (shown at *λ* = 550 nm).

The wavelength dependency of the BRDF has been captured with varying success by different BRDF models which are included in OBL as well. Cook and Torrance described a physics based BRDF model which can capture much of the spectral dependency of roughened metals [14]. Lafortune et al. approximated the measured BRDF of materials using a summation of a series of cosine lobes [15]. Although an approximation, their model provided a simple means of capturing much of the detail of the BRDF, including color dependency. Sample BRDF lobes from these two OBL models at a single wavelength are shown in Fig. 3.

**Fig. 4:**
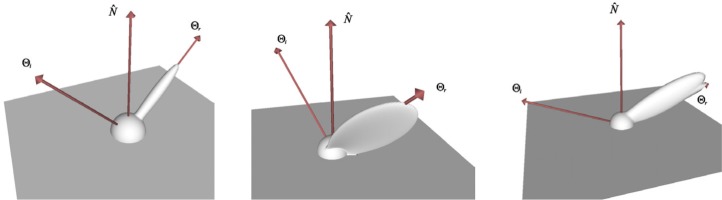
Three BRDFs used to model first-surface reflection: Phong, Ward, and He.

Several BRDF models included in OBL have also been developed to capture the shininess of materials. Phong proposed a cosine function raised to a power to approximate this type of surface [16]. Ward fit a Gaussian based function to measured data, and included the ability to model anisotropic effects [17]. He et al. derived a physics based BRDF for shiny surfaces which incorporates Fresnel reflectance and wave optics [18]. Examples of the BRDF models are shown in Fig. 4.

The Oregon BRDF Library which includes the above BRDF models and more has been used as the BRDF interface for a BRDF visualization system. A second tool was also created to determine the correspondence between the BRDF models of OBL and standard appearance scales. These tools have been used to provide the BRDF values for realistic image synthesis.

## 3. NEFDS

The Beard-Maxwell model [8] is another physics based BRDF model, but one whose parameters are specified with empirical measurements. The model is especially important because there is a government database of surface reflection parameters, the Nonconventional Exploitation Factors Data System [19] (NEFDS), that utilizes a modified form of the Beard-Maxwell model, the NEF Beard-Maxwell (NEF-BM) model. Additional surface reflection parameters for the database can be determined because a measurement protocol, using existing radiometric instruments, has been specified [7].

### 3.1 Beard-Maxwell Reflection Model

The Beard-Maxwell model presented by Maxwell et al. [8] is built on the assumption that the material surface is a three dimensional terrain of micro-facets of varying orientation. In this model, reflected light is the result of only two physical occurrences. Light is reflected off one of the micro-facets (first surface reflectance) and light is scattered out of the surface after having first entered the sub-surface medium (volumetric reflectance).

**Fig. 5:**
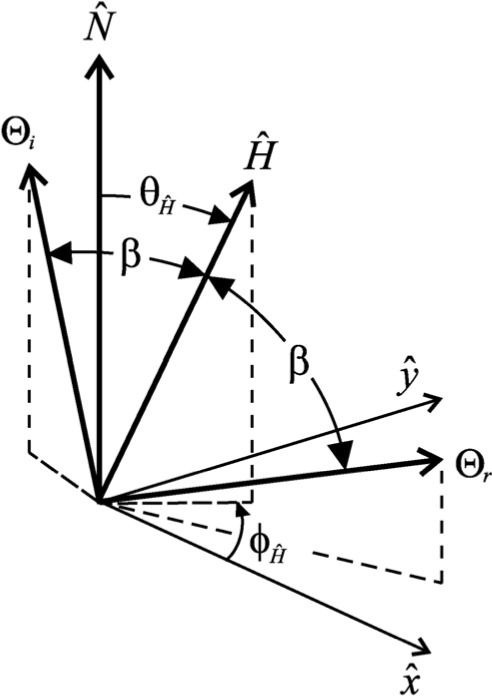
Beard-Maxwell BRDF model geometry.

First surface reflection causes light to be reflected in the specular direction (i.e., mirror reflection) off each individual micro-facet as determined by the micro-facet’s normal rather than the macro-surface normal. Therefore the distribution of the first surface reflectance is determined by the distribution of the micro-facet normals which in turn is driven by the density function *Ξ* (***Θ***), the relative density of micro-facet normals (per steradian) in vector direction ***Θ***. Maxwell et al. calculated the first surface reflectance to be

$$\rho_{\text{fs}}\left(\Theta_{i},\Theta_{r}\right)=\frac{R\left(\beta \right)\Xi \left(\hat{H}\right)}{4\cos\theta_{i}\cos\theta_{r}}SO$$ (3)

where *Ĥ* is the half angle vector, *β* is the bistatic angle (i.e., the angle between either the incident or reflected direction and the half angle vector), *R*(*β*) is the Fresnel reflectance, and *SO* is a shadowing and obscuration term. Figure 5 is a diagram of the geometry used by the Beard-Maxwell model.

Rather than attempting to measure *Ξ* of Eq. (3) directly, Maxwell et al. replaced it with the measured zero-bistatic (*β* = 0) first surface reflectance at the half angle, *ρ*_fs_(*Ĥ,Ĥ*). *SO* = 1 when *β* = 0, so Eq. (3) can be rewritten as

$$\Xi \left(\hat{H}\right)=\frac{4\rho_{\text{fs}}\left(\hat{H},\hat{H}\right)\cos^{2}\theta_{\hat{H}}}{R\left(0\right)}$$ (4)

This is simplified further by their assumption that the surface is isotropic. In that case to generate reflectance values for all incident and reflected directions, *ρ*_fs_(*Ĥ,Ĥ*) need only be sampled at the angles $0\leq \theta_{\hat{H}}\leq \frac{\text{π}}{2}$ and the azimuthal angle *ϕ_Ĥ_* = 0.

Light reflected from the first surface is assumed to maintain its original polarization (i.e., incident and reflected light are of like polarization) while any light reflected through volumetric scattering is assumed to be totally depolarized. In this way, separation of the first surface reflectance from the volumetric reflectance can easily be performed by measuring the polarization of reflected light. A few additional, well defined measurements are used to determine other model parameters, such as the surface index of refraction and the shadowing and obscuration parameters.

### 3.2 Database System

At the heart of NEFDS is a database system containing the BRDFs of over four hundred materials. The materials in the database fall into 12 different categories: asphalt, brick, camouflage, composite, concrete, fabric, water, metal, paint, rubber, soil and wood. This database which can be accessed either through the interactive XWindows program *NefMenu*, or by using command line control, allows the user to query for BRDF values of materials or material groups at ranges of wavelengths for any given geometry.

**Fig. 6:**
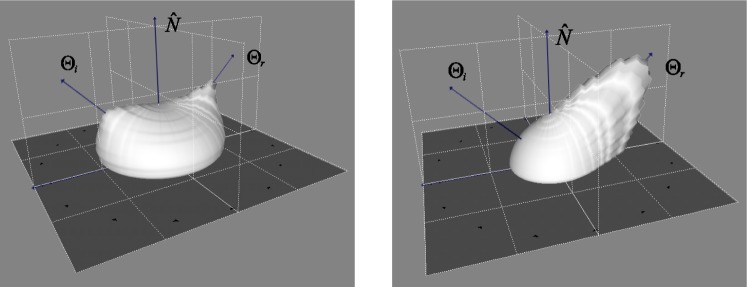
BRDFs of cement (left) and bare construction lumber (right) obtained from NEFDS.

The variation of material BRDFs available through NEFDS can be seen in Fig. 6, which shows the BRDFs of cement and lumber. Notice the significant difference in geometry that can be characterized by the modified Beard-Maxwell reflection model. This strength (the ability to capture a wide variety of BRDF distributions) coupled with the systematic method of measurement makes for a very powerful tool.

Although the number of materials is large and the variety is wide, the selection is limited by two key conditions. One requirement for the inclusion of a material in the database is that the BRDF of the material must be well represented by the NEF-BM BRDF model. For example, the BRDF should not be characterized by strong surface anisotropy since the NEF-BM BRDF model only works with isotropic data. There are in fact some materials included in the NEFDS which are anisotropic. To represent anisotropic material using the NEF-BM model, the material’s BRDF is averaged to isotropy.

The second condition is a result of the main application of NEFDS—materials were selected to be part of the database based on their relevance to the field of remote sensing. These materials are mostly objects which would be viewed from a remote sensor, such as a satellite. However, the well defined measurement protocol lends to the future inclusion of other materials.

A potential limitation of the NEFDS is that the modified Beard-Maxwell equation used with NEFDS results in inaccuracy at grazing angles. Light incident at grazing angles will result in more energy reflected than was incident. In the application to remote sensing this is not relevant since measurements are performed far enough away from grazing, but it might be relevant in the application to computer graphics. In practice, the inaccuracy usually has not resulted in noticeable artifacts, primarily because of the foreshortening of incident energy which also occurs at grazing angles. However, this is an important point to keep in mind.

## 4. iBRDF

The BRDF models represented in the previous two sections can capture subtle differences in surface light reflection. However most shaders, the algorithmic implementation of the reflection model, are not able to represent the BRDF at this level of generality. In order to use these BRDFs in generating synthetic images requires a shader capable of capturing the detail which is available in the BRDFs. A new shader, called iBRDF, has been developed which accurately simulates this detail through its ability to utilize any arbitrary BRDF function.

### 4.1 Alias Method

iBRDF was implemented within the Radiance Lighting Simulation and Rendering System (Radiance) [6, 20, 21] to generate synthetic images. Radiance is a suite of programs built around an advanced distributed raytracer designed for realistic image synthesis. It was selected because it is a physically-based rendering system designed to accurately model the light behavior of a scene using physical units [21]. Using such a system complements the validity of the results obtained by the physics based BRDFs within OBL and those generated from NEFDS. Additionally, the source code to Radiance is publicly available [6] and the program is currently in wide use, aiding future work.

Radiance is a distributed ray tracer which utilizes Monte Carlo importance sampling to solve Eq. (1), termed the rendering equation in computer graphics [22]. The rendering equation specifies the reflected radiance in direction ***Θ***_r_ from the values of the surface’s BRDF and the incident irradiance, integrated over all incident directions. As mentioned earlier the solution of this integral often is the most computationally expensive task of a rendering program. For this reason the solution to this integral is often found through the use of Monte Carlo integration.

We have developed an efficient method of performing this Monte Carlo integration. Instead of casting rays in a uniform distribution about the hemisphere and weighting the returned value by the reflectance, the ray distribution itself is weighted by the reflectance. This can be done in a straightforward manner when the BRDF is composed of invertible functions such as Gaussians. When the BRDF is represented discretely, either by taking measurements over the hemisphere or by sampling a non-invertible functional form, another method must be used to generate random variates for Monte Carlo integration. This can be accomplished by first subtracting the smallest hemisphere that fits within the BRDF data. This removes the diffuse or uniformly varying portion of the BRDF and leaves only the highly directional specular part. The alias selection method [23] can be employed to create random variates from these remaining specular reflectances

**Fig. 7:**
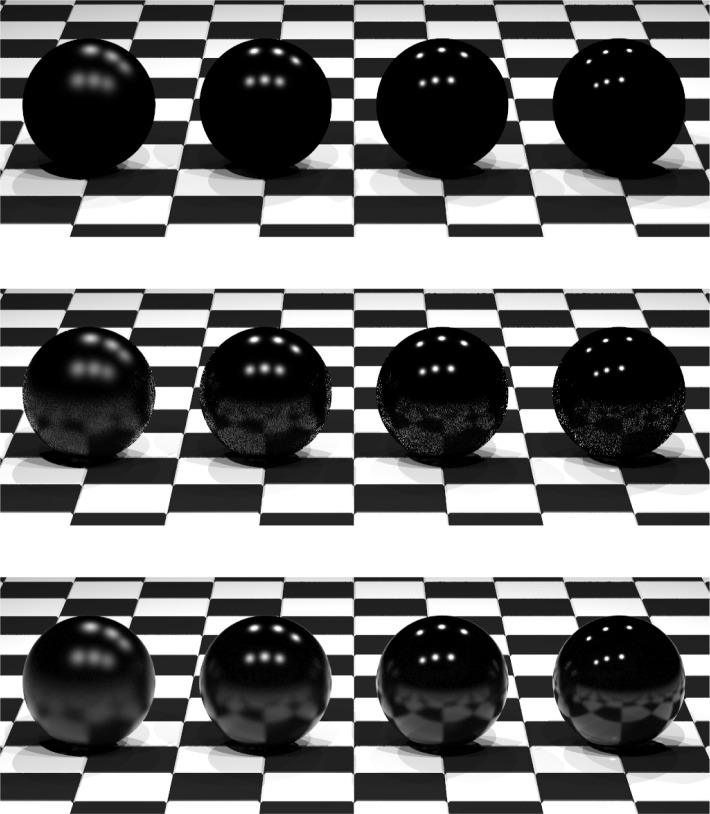
Four spheres of increasing glossiness rendered using three different methods. Top—Radiance’s built-in arbitrary BRDF shader incorrectly ignores the contribution from indirect illumination. Middle—Uniform Monte Carlo sampling (160 samples per pixel) results in an image filled with sampling noise. Bottom—Monte Carlo importance sampling with iBRDF (160 samples per pixel) correctly renders image.

Radiance was designed with built-in support of arbitrary BRDFs, but only for computing the direct contribution of the dominant light sources. In Radiance, the dominant light sources are handled separately to reduce the variance introduced in the Monte Carlo evaluation of Eq. (1). Figure 7 (top) which uses the built-in BRDF shader, shows the direct reflection of the six light sources correctly, but there is no reflection at all of the indirect illumination from the surrounding checkered floor. Performing uniform sampling of the BRDF begins to capture this indirect contribution from the floor as seen in Fig. 7 (middle), but the reflected image of the floor contains excessive noise. The best results are obtained with the importance sampling of iBRDF in Fig. 7 (bottom). The reflection of the floor is accurately captured in the four spheres of this image using the same number of samples as the middle image.

### 4.2 Application of iBRDF

#### 4.2.1 Coated Epoxy

**Fig. 8:**
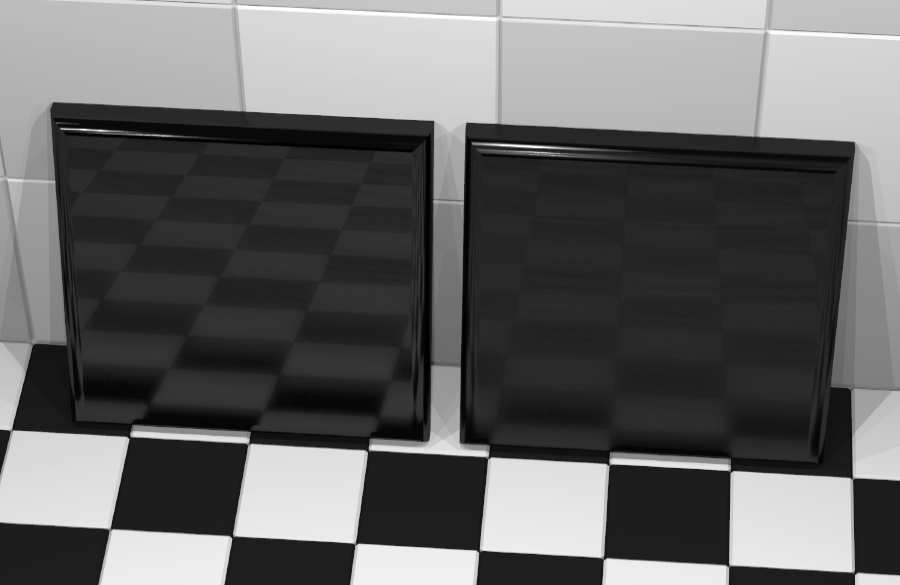
Rendering from reflectance data generated using the Ray method and a surface topographical map of coated epoxy samples with rms roughness values 201 nm (left) and 805 nm (right).

iBRDF was used to render reflectance data coming from samples of black glass covered with a layer of clear epoxy. In this simple first case, the surface was isotropic and a pre-specified degree of surface roughness was obtained by appropriate fabrication conditions. We were interested in seeing how well differences in the rendered images of samples with different roughnesses displayed the gloss differences in the samples. Black glass minimized the effect of subsurface reflection and transmission, and the index of refraction of the epoxy and the glass were approximately equal. Details on sample preparation, reflectance modeling, and measurement can be found in [24]. The reflectance modeling makes use of surface topography measurements—an approach that is new in the rendering field. The reflection model known as the ray method, is based on the assumption that incident light is specularly reflected by the local tangent plane on the surface. Measurements of local surface height were made using scanning white light interferometry by researchers in the Manufacturing Engineering Laboratory (MEL) who then used these heights to construct the normals to local tangent planes needed to compute the scattering directions. The BRDF was computed by simulating the uniform illumination of the surface for various incident directions, and counting the number of scattered rays that reach detectors distributed over a hemisphere of scattering directions. Comparisons between the results of this calculation and optical measurements of the sample and comparison with a more rigorous scattering model showed that the ray method furnished a good approximation to the BRDF. The method is also computationally efficient so a sufficient number of incident and scattering directions could be quickly generated and used as input for iBRDF. Figure 8 shows two black glass tiles. The tile on the left has rms roughness of approximately 0.2 μm while the tile on the right has approximate roughness of 0.8 μm. The images were consistent with visual inspection of the tiles. The study demonstrated that gloss loss due to surface roughening could in fact be predicted by the rendering. This work is reported in [25].

#### 4.2.2 NEFDS

**Fig. 9:**
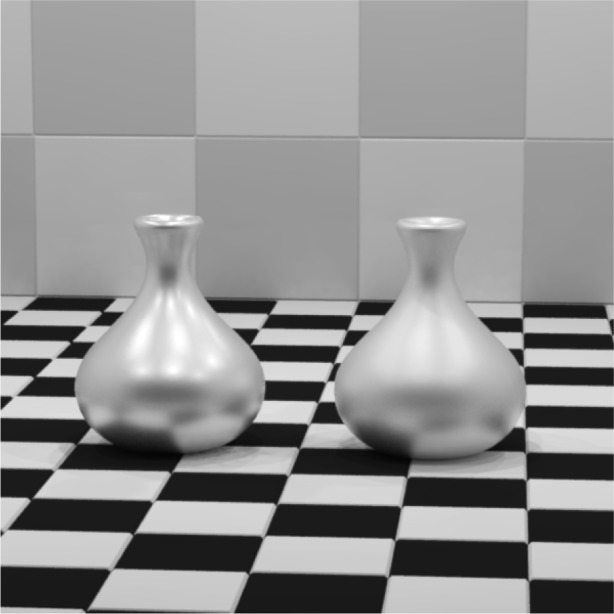
Simulation of coarse and fine metallic paint on vases.

As discussed in Sec. 3, not only does NEFDS offer hundreds of pre-existing materials, it also allows new materials to be added. Using the methods of measurement referred to in that section, two surfaces painted with gray metallic paint were fit to the NEF-BM model. The surfaces were fabricated by automotive coating processes and were measured at NIST [9]. The paints were mixed so that one had mostly coarse metallic flakes while the other was dominated by fine metallic flakes. The paint with a greater number of fine flakes had a larger diffuse component due to more edge scattering. This is easily captured by the NEF-BM model and iBRDF as can be seen in the rendered image of Fig. 9.

#### 4.2.3 ASTM Standards

While it is important to be able to accurately depict the full BRDF of a material, there is also much merit in the ability to characterize a material with appearance attributes such as gloss or haze. To this end, people conducting research in appearance have sought to develop and standardize a number of simple measurements and corresponding measuring devices which easily and objectively quantify the reflection properties of a surface. The result is a number of one-dimensional scales of appearance, such as gloss and haze, and inexpensive appearance measurement devices, such as glossmeters.

The standard specular gloss measurement defined by the American Society for Testing and Materials (ASTM) in ASTM method D523 measures the magnitude of light reflected in a small solid angle about the specular direction [26]. ASTM method E430 specifies that haze is a measure of the fraction of light reflected in an off-specular direction to that reflected in the specular direction [27]. These well defined measurements result in a single numerical value describing particular appearance attributes of the measured surface. An analogous calculation may be performed on the BRDF of a surface through computer simulation of the measurement protocol. In this way, a simulated glossmeter or hazemeter can be used to determine the gloss or haze of any arbitrary BRDF.

**Fig. 10:**
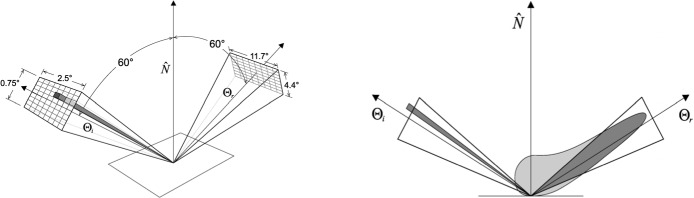
Left—Subdivision of light meter apertures using the 60° specular gloss specifications [26]. The source and receptor apertures are oriented in directions ***Θ***_i_ and ***Θ***_r_, 60° down from the surface normal, $\hat{N}$, in the plane of incidence. Right—Flux passing through receptor aperture due to one source aperture subdivision. Aperture sizes are not to scale.

A computer program was developed which applies the measurement protocol of standardized appearance tools to BRDFs in order to simulate their results [28]. This new virtual light meter is essentially a customized integration tool, using numerical quadrature of the specified BRDF model over an adaptively subdivided source and receptor aperture (Fig. 10) to compute the final standard appearance value. In addition to being able to calculate the current standards, the virtual light meter can be customized for other measurements. The customizable parameters include the size and locations of the source and receptor apertures, the specular angle, the surface orientation, and the reflection model.

The reflected flux passing through the receptor aperture is directly responsible for the standard gloss and haze values. Integration of this flux begins by first subdividing the source aperture. For each sampled point on the source aperture, the receptor aperture is adaptively subdivided. Subdivision of the receptor aperture continues until either the discretely computed flux approaches some stable value or a maximum subdivision depth is reached. Figure 10 is an example of the flux passing through the receptor aperture resulting from a single subdivided source element. After the flux due to this source element is computed, the process is repeated for the other source elements. Adaptive subdivision of the source continues until either the flux approaches some stable value or a maximum subdivision depth is reached.

**Fig. 11:**
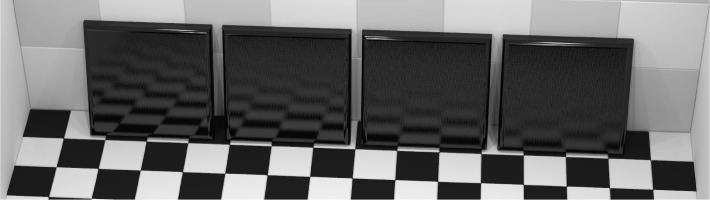
Tiles with measured 20° specular gloss values 80, 60, 40, and 20.

**Fig. 12:**
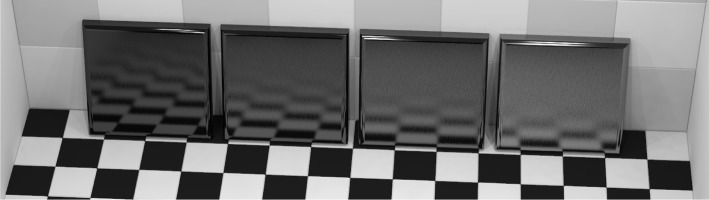
Tiles with measured 2° haze values 10, 60, 110, and 160.

This program was used with surface reflection properties defined by the Ward reflection model [17] in order to develop a correspondence between the model parameters and the standard 20° gloss values. Using this correspondence, an image of tiles with decreasing gloss was generated (Fig. 11). These tiles, with model parameters selected to produce 20° gloss values of 80, 60, 40, and 20, create a close correspondence between appearance and gloss value. In a similar fashion, the correspondence between 2° standard haze and the Ward model was determined and used to render four tiles of increasing haze (Fig. 12). The BRDF model roughness parameter values were chosen so as to produce 2° haze values for these tiles of 10, 60, 110, and 160. The 20° gloss of the four haze tiles was maintained at 100 by scaling the specular coefficient.

#### 4.2.4 Metallic Paint

A more recent appearance measurement, currently in the standardization process [29], is that used to characterize metallic paints and plastics. These metallic surfaces contain small platelets of metal usually accompanied by colored particles or dyes in the substrate. The platelets are oriented nearly parallel to the surface so as to create a strongly directional reflection. This directionally reflected light changes color as it passes through the colored substrate.

The method proposed for standardization specifies measurement of the tristimulus values at three angles: near specular, far from specular and one more angle in between. Interpolation of these three measured values has been found to accurately characterize the appearance of metallic surfaces. A BRDF can then be generated from the interpolated tristimulus values and used with iBRDF to render synthetic images of objects modeled with metallic paint.

**Fig. 13:**
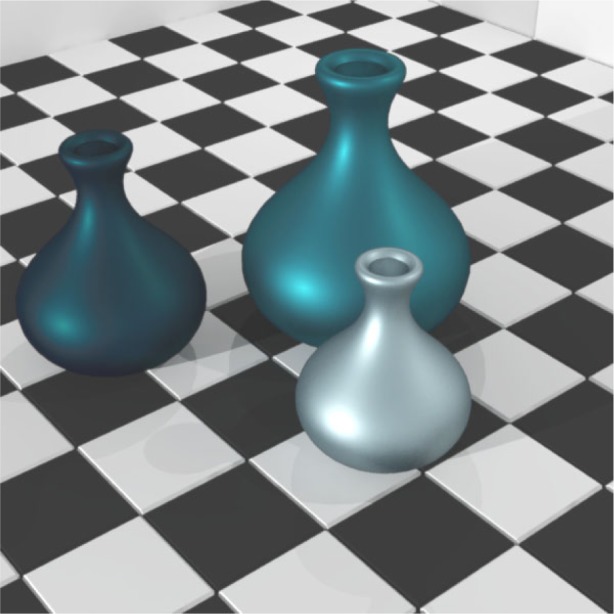
Three vases with metallic paint but no clear coat.

**Fig. 14:**
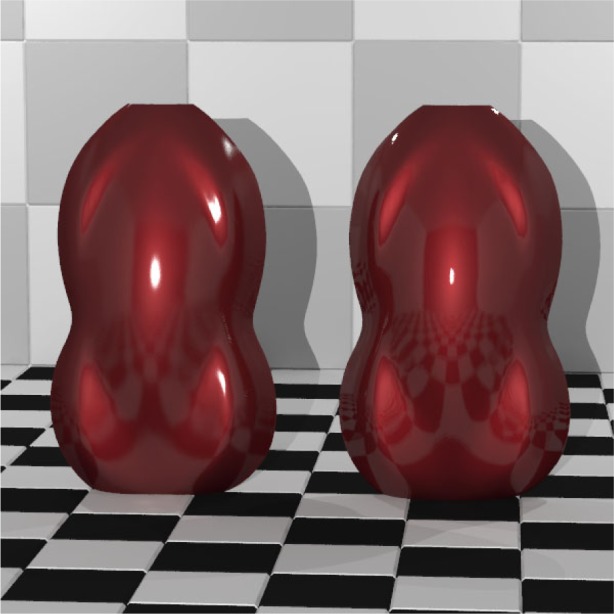
Two automotive shells with 20° specular gloss of 10 and 60.

Figure 13 is an image of three vases modeled using tristimulus data measured from actual metallic paint samples and rendered using iBRDF. These vases demonstrate the sub-surface characteristic of metallic paint but not the usual glossiness attributed with metallic automobile finish. Combining the gonio-apparent sub-surface reflection with a first-surface BRDF leads to a more realistic image as can be seen in Fig. 14. The first-surface BRDF was chosen so that the 20° gloss values are 10 for the left shell and 60 for the right.

## 5. Conclusion

In this work we reported the results of our efforts to create visually and radiometrically accurate renderings of the appearance of gloss in coated epoxy samples (with controlled roughness), gloss and haze in painted metallic panels, and color in painted metallic panels. These investigations help build a path from the material properties of coatings to a visually accurate representation of their appearance. To achieve this we had to link BRDF measurement and modelling on the one hand, and computer graphic rendering on the other. We started with a publicly available program Radiance that had a built-in reflectance model that is accurate for so-called Gaussian surfaces that are well described by Gaussian statistics. Its major drawback was that it could not accept measurement data as input and could not handle non-Gaussian surfaces. The enhanced rendering program iBRDF constructed by Westlund and Meyer, does accept arbitrary input because it performs Monte-Carlo inversion for arbitrary distributions and therefore removes the restriction to the kind of BRDFs used in Radiance. We found that rendering required BRDF models where a large number of values could be produced with computational efficiency. Our approach to measurement and modelling was designed to achieve the balance between this need and physical accuracy.

iBRDF was used to render images of coated black glass samples from reflectance data. Using a reflectance model based on the distribution of normals to local tangent planes on the surface, a BRDF was constructed from surface topographical measurements. In the painted metallic panel case a modified Beard-Maxwell reflectance model was used. This model is physically based but has terms and parameters that can be determined by a measurement protocol as was used in the NEFDS database. M. Nadal of the Physics Laboratory performed the measurements on the panels.

ASTM gloss measurement standards are linked to the visual evaluation of gloss. Rendering is a potentially important tool in developing such standards or could become a procedure cited in new gloss standards. The work described in Sec. 4.2.3 is a first step along this line. Rendering materials under an ASTM standard procedure will enable the systematic study of the variation of the appearance of standard materials with distance between object and viewer or degree of illumination. Moreover this work shows how comparisons can be made between different rendering programs so that a computer graphic based gloss standard can be defined independently of the program used. We also note that programs rendering a set of objects with defined gloss standards can be compared, thus giving rise to a possible standard for rendering programs.

An important outcome of our work has been the demonstration that satisfactory rendering of many colored objects can be done with just a few measurements. This was seen in the rendering of metallic painted shells using iBRDF.

The BRDF has played an essential role in our study of appearance but progress in capturing complex visual effects will have to go beyond this function. The glittering micro-appearance of metallic paint so evident when one approaches a newer automobile is an effect that involves scales that are too small to be described by BRDF and depends on human binocular vision as well. Image texture is an extremely important attribute and it too falls outside the range of BRDF description. We have also neglected the effects of paint application in our modeling. Nevertheless, the rapid developments in computer graphics—where the turn around time between theoretical formulation and practical application is so short—make significant progress on these problems in the near future an exciting and likely possibility.
